# Detection of FLT3 Oncogene Mutations in Acute Myeloid Leukemia Using Conformation Sensitive Gel Electrophoresis

**DOI:** 10.3390/ijms9112194

**Published:** 2008-11-11

**Authors:** Mamdooh Gari, Adel Abuzenadah, Adeel Chaudhary, Mohammed Al-Qahtani, Huda Banni, Waseem Ahmad, Fatin Al-Sayes, Sahira Lary, Ghazi Damanhouri

**Affiliations:** 1 Medical Technology Department, Faculty of Applied Medical Sciences, Genomic Medicine Unit, Center of Excellence of Genomic Research. P. O. Box 80216, King Abdulaziz University-Jeddah, Kingdom of Saudi Arabia. E-Mails: aabuzenadah@kau.edu.sa (A. A.); adeel.gc@gmail.com (A. C.); mhalqahtani@kau.edu.sa (M. A.); hbanni@kau.edu.sa (H. B.); 2 Hematology Department, Faculty of Medicine, King Abdulaziz University- Jeddah, Kingdom of Saudi Arabia. E-Mails: fatinsayes@yahoo.com (F. A.); ghazid@zajil.net (G. D.); 3 Biochemistry Department, Faculty of Sciences, King Abdulaziz University- Jeddah, Kingdom of Saudi Arabia. E-Mail: slary@kau.edu.sa (S. L.)

**Keywords:** AML, Flt3, ITD, CSGE, Mutational analysis

## Abstract

FLT3 (fms-related tyrosine kinase 3) is a receptor tyrosine kinase class III that is expressed on by early hematopoietic progenitor cells and plays an important role in hematopoietic stem cell proliferation, differentiation and survival. FLT3 is also expressed on leukemia blasts in most cases of acute myeloid leukemia (AML). In order to determine the frequency of FLT3 oncogene mutations, we analyzed genomic DNA of adult *de novo* acute myeloid leukemia (AML). Polymerase chain reaction (PCR) and conformation-sensitive gel electrophoresis (CSGE) were used for FLT3 exons 11, 14, and 15, followed by direct DNA sequencing. Two different types of functionally important FLT 3 mutations have been identified. Those mutations were unique to patients with inv(16), t(15:17) or t(8;21) and comprised fifteen cases with internal tandem duplication (ITD) mutation in the juxtamembrane domain and eleven cases with point mutation (exon 20, Asp835Tyr). The high frequency of the flt3 proto-oncogene mutations in acute myeloid leukemia AML suggests a key role for the receptor function. The association of FLT3 mutations with chromosomal abnormalities invites speculation as to the link between these two changes in the pathogenesis of acute myeloid leukemiaAML. Furthermore, CSGE method has shown to be a rapid and sensitive screening method for detection of nucleotide alteration in FLT3 gene. Finally, this study reports, for the first time in Saudi Arabia, mutations in the human FLT3 gene in acute myeloid leukemia AML patients.

## 1. Introduction

The FLT3 gene (fms-like tyrosine kinase), is a member of the class III tyrosine kinase receptor family that includes c-kit, c-fms and the platelet-derived growth factor receptors (PDGFRs) [1–3]. Class III receptor tyrosine kinases (RTKs) share sequence homology and have a similar overall structure, with five immunoglobulin-like repeats in the extracellular domain, a single transmembrane domain (TM), a juxtamembrane domain (JM), two intracellular tyrosine kinase domains (TK1 and TK2) divided by a kinase insert domain (KI), and a C-terminal domain [4]. The genomic locus encoding the FLT3 receptor has recently been shown to comprise 24 rather than the expected 21 exons, ranging 83–562 bp [5]. Data from different sources suggest a pathogenic role of FLT3 in acute myeloid leukemia (AML). Hence functional analysis of FLT3 expression has been documented in many cases of AML, besides of immortalized human myeloid and monocytic cell lines [3, 6–8]. The ITD and activation loop mutations of the receptor have resulted in the constitutive FLT3 kinase activity and FLT3 receptors harboring such mutations when introduced into mammalian cells downstream signaling pathways lead to factor-independent growth *in vitro* and leukemogenesis *in vivo* [9, 10]. Thus the production of FLT3 mutant protein in primary murine bone marrow cells induces a lethal myeloproliferative phenotype [11]. It is known that FLT3 is a leukemia oncogene and activating FLT3 mutations are likely to contribute in the development of leukemia in humans.

In addition, several small molecule inhibitors have also been implicated in blocking the kinase activity of FLT3 effectively [11–14]. These can prolong the life span of mice harboring leukemia expressing mutant FLT3 receptors [11, 15]. In clinical trials, FLT3 inhibitors reduced FLT3 phosphorylation [16–18], and lowered leukemia blast counts in patients with advanced therapy-refractive AML [18, 19].

Until now, no study has reported the frequency and prevalence of FLT3 mutations in AML patients in the Kingdom of Saudi Arabia. This study was conducted with that objective in mind and was therefore undertaken using polymerase chain reaction-conformation sensitive gel electrophoresis (PCR-CSGE) on DNA extracted from archival bone marrow of Saudi AML patients.

## 2. Results and Discussion

### 2.1. Detection of the FLT3-ITD mutation

**Figure 1.**
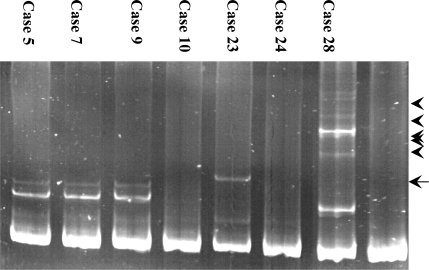
CSGE analysis of exons 14 and 15 PCR product amplified from AML patients. CSGE gel demonstrating abnormal patterns (indicated by arrowheads) compared to normal pattern (lane N, PCR product amplified from healthy individual, indicated by arrow).

**Figure 2.**
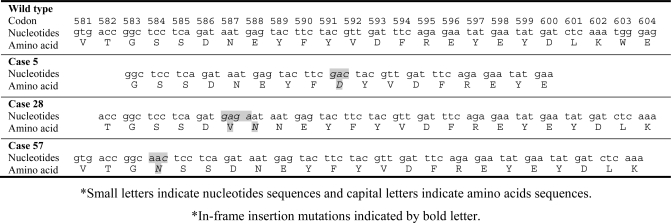
Sequence analysis of exons 14 and 15 of FLT3 gene. Inserted nucleotides for tandem duplications of the Flt3 gene observed in AML cases with apparent CSGE patterns.

In order to screen for the FLT3-ITD mutation, exons 14 and 15 of the FLT3 gene were amplified from genomic DNA of 129 AML patients using PCR, followed by conformation sensitive gel electrophoresis (CSGE) analysis. Abnormal CSGE patterns in 15 AML patients were identified in PCR fragments and the remaining patients reported no such patterns. These abnormal patterns, shown in Figure 1, were due to conformational changes occurred in the gel indicating nucleotide alteration (in-frame insertion mutation) within the PCR fragment. When direct DNA sequencing analysis was carried out on all 15 AML cases with abnormal CSGE patterns, ITD mutations were detected in all cases with lengths varying between 24–60 bp. The FLT3-ITD mutations detected included either a part or whole stretch of tyrosine-rich sequence of the FLT3 gene located between codons 589–599 (Figure 2). Furthermore, these mutations were located in-frame of the JM domain of FLT receptor which gave the evidence of tandem duplications, thus confirming the ITD in the samples.

### 2.2. Detection of the Asp835Tyr mutation

**Figure 3.**
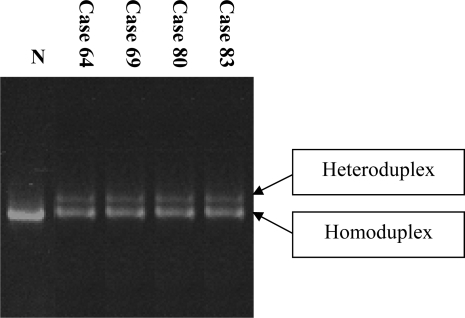
CSGE analysis of exon 20 of FLT3 gene demonstrating abnormal CSGE pattern compared to that seen in a normal individual (Lane N).

**Figure 4.**
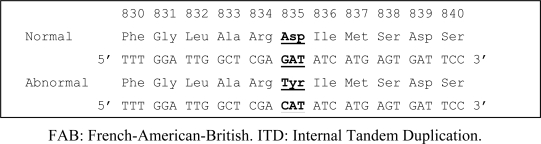
Point mutation in exon 20 of FLT 3 gene in AML cases detected by direct DNA sequence analysis. Mutated nucleotides are represented by changed amino acid, (bold).

In addition to the FLT3-ITD mutation, the Asp835Tyr mutation is also prevalent in AML cases. To screen our cohort for the presence of this mutation, exon 20 of the FLT3 gene was subjected to PCR-CSGE followed by direct sequencing in all 129 AML cases. Eleven cases of AML (8.5%) exhibited an abnormal CSGE pattern (Figure 3), and sequencing revealed a G to C mutation in codon Asp835Tyr (Figure 4). Six of these were classified as AML M4, four of which demonstrated inv(16). In addition, FLT3 ITD mutations were identified in 15 patients; however, no case possessed both an ITD and Asp835 mutation jointly. The detailed clinical characteristics of AML patients forming the basis of this observation are summarized in Table 1.

### 2.3. Analysis of correlation with survival

**Figure 5.**
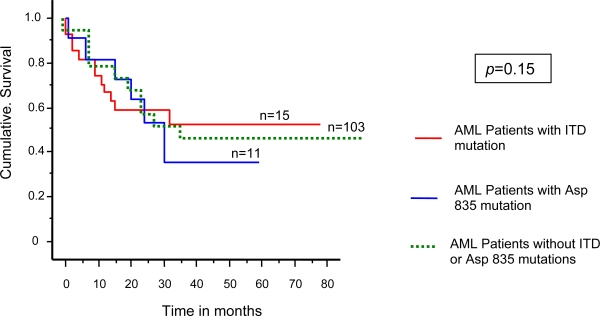
Kaplan-Meier cumulative survival analysis for AML patients with Asp 835 and ITD mutations and AML patients without mutations. Blue line indicates patients with Asp 835 mutation, red line indicates patients with ITD mutations and green line patients without flt3 mutations. The survival analysis showed no significance differences between the three groups (*p* = 0.15).

Survival analysis was calculated using Kaplan-Meier plot for comparison between Asp835Tyr and ITD AML patients. The survival curves were plotted for AML patients with Asp 835 mutation (n = 11) and ITD mutations (n = 15). The Mantel-Cox test demonstrated that the difference between the two groups of AML patients was not statistically significant (Figure 5).

We undertook in this study the analysis of ITD and Asp 835 mutations of the FLT3 gene in Saudi AML patients in order to better understand the prevalence of these mutations in our cohort of cases. FLT3-ITD mutation is thought to cause the constitutive and ligand-independent activation of the receptor, but Fenski *et al.* [20] have shown that FLT3 oncogene is constitutively activated in 3/18 cases of AML patients and only 1/3 of those cases exhibited the FLT3-ITD mutation. It was therefore suggested that some other mechanisms are operational for the activation of the receptor, either because of mutations elsewhere in the gene or due to autocrine mechanisms. This prompted us to include in our screen exon 20 and identify mutations involving Asp 835. Previous studies indicated that Asp 835 highly conserved homologues, are probably playing a crucial role in the function of the activating loop of the JM region and kinase domain of class III RTKs [20–26]. The frequency of FLT3-ITD mutation identified in this study is much lower than reported for other ethnic populations which range between 20%–25% in Japanese [27], Chinese [28], Thai [29] or German [30] populations. It is interesting to find that all the detected cases of FLT3-ITD mutations exhibited cytogenetic aberrations, in particular t(15;17), t(8;21) and Inv(16). These chromosomal rearrangements are commonly found in adult AML cases as in our cohort inv(16) is the most common event at 26.3%, followed by the presence of t(15;17) translocation (17.8%). The t(8;21) translocation constituted only 8.5% of the cases in our cohort. The FLT3-ITD mutations were all detected in cases with cytogenetic translocations. Whether this reflects a case of global genomic instability or not is unknown. Screening of a larger cohort of samples may shed some light into the potential association between FLT3-ITD mutations and genomic instability.

The first aim of this study was to screen the FLT3 gene for previously reported mutations in AML using a sensitive and simple method to determine the frequency of the mutations. Efficiency and sensitivity were the main criteria to be considered in the selection of the method of mutation detection to be used in the analysis of the FLT3 gene. CSGE was the method of choice for screening the FLT3 gene because of its high sensitivity and simplicity as reviewed by Ganguly [31]. The technique has been used in different studies for the analysis of factor VIII gene in hemophilia A patients [32–33] and other genes including the von Willebrand factor (VWF) gene [34], factor IX [35]. For example, the sensitivity of CSGE in the detection of mutations was 100% in 21 hemophilia B patients [35] and 88% in patients with VWD [34]. Moreover, CSGE was selected as the method of choice to ensure all previously known FLT3 mutations can be detected.

## 3. Conclusions

In conclusion, we report mutations in the human FLT3 gene in a cohort of Saudi AML patients. These mutations are likely to lead to constitutive activation of the FLT3 receptor. The FLT3-ITD mutation rates reported in this study are lower than expected which would justify that future studies will need to screen the complete coding and regulatory sequence of the FLT3 gene to determine whether additional mutations are associated with the pathogenesis of myeloid malignancy.

## 4. Experimental Section

### 4.1. Patient DNA samples

Sample DNA was collected from 129 routinely-processed unstained bone marrow slides diagnosed as Acute myeloid leukemia (AML), which had been stored in a box at room temperature in an air-conditioned storage room for up to 20 years (from 1987 to 2007) in the archives of Hematology Laboratory at King Abdulaziz University Hospital. Genomic DNA was extracted (as optimized by Gari, *et al.* [36]) from the scraped material of each bone marrow slide using the Nucleon BACC1 DNA extraction kit (Nucleon Biosciences). Theses samples of genomic DNA were selected at presentation from 129 cases of AML (53 women, 76 men, and mean age 40.3 years, range 22–65). The cases were classified according to the FAB criteria [37] as M0 (n = 5), M1 (16), M2 (n = 38), M3 (n = 23), M4 (n = 34), M5 (n = 9) and M6 (n = 4). Sixty-eight patients had good risk and 61 had intermediate risk disease, as defined by standard cytogenetic analysis [38]; inv(16) (n = 34), t(15;17) (n = 23) and t(8;21) (n = 11).

### 4.2. Genomic DNA amplification

DNA amplification was performed by polymerase chain reaction (PCR). All exons, and intron/exon boundaries of the FLT3 gene involved in this study were amplified independently. The entire coding sequence and intron/exon boundaries corresponding to exons 14, 15 (ITD) and 20 (Asp835) of the FLT3 gene were amplified independently, using oligonucleotide primers as shown in Table 2.

The PCR reactions contained the following: genomic DNA (500 ng), (NH_4_)_2_SO_4_ (16.6 mM), Tris HCL (pH 8.0, 67 mM), β-mercaptoethanol (10 mM), bovine serum albumin (BSA, 100 μg), each primer (300 ng), dNTPs (200 μM), MgCl_2_ (1.50 mM), and Taq DNA polymerase (1 U) in a final volume of 50 μL. Samples were initially denatured at 94^°^C for 5 minutes. DNA amplification was performed by 35 cycles of denaturation at 94^°^C for 1 minute, annealing at 55^°^C for 30 second and extension at 72 ^°^C for 30 second. Five μL of each PCR products was loaded onto 5% polyacrylamide gel or agarose gel to ensure the amplification had occurred.

### 4.3. Mutation detection

Conformation sensitive gel electrophoresis (CSGE) was used to screen for mutations as described in [31]. Briefly, PCR products were denatured by heating to 95^°^C for 5 minutes and then incubated at 65^°^C for 30 minutes. These PCR products were resolved on 10% polyacrylamide gels; 99:1 ratio acrylamide (BDH):bis-acrolypiperazine (BAP; Fluka), 10% ethylene glycol (Sigma), 15% formamide (Sigma) and 0.5 × TTE buffer (1 × TTE = 89 mM Tris, 28.5 mM taurine, 0.2 mM EDTA). Samples displaying abnormal CSGE profiles compared to that obtained from a normal individual were directly sequenced using automated DNA sequencer (API 310 prizm).

## Figures and Tables

**Table 1.** Clinical characteristics of AML cases and accompanied mutations.

Case no.	Age (years)	Sex	FAB subtype	Karyotype	Mutations
5	73	F	M3	46;XY, t(15;17)	ITD
7	32	M	M3	46;XY, t(15;17)	ITD
9	48	F	M3	46;XY, t(15;17)	ITD
12	51	F	M2	46;XY, t(8;21)	ITD
18	24	F	M3	46;XY, t(15;17)	ITD
20	62	F	M3	46;XY, t(15;17)	ITD
23	32	M	M4	46;XY, inv(16)	ITD
25	33	M	M4	46;XY, inv(16)	ITD
28	38	M	M3	46;XY, t(15;17)	ITD
29	46	F	M2	46;XY, t(8;21)	ITD
41	48	M	M3	46;XY, t(15;17)	ITD
44	62	F	M3	46;XY, t(15;17)	ITD
49	64	F	M4	46;XY, inv(16)	ITD
53	43	F	M2	46;XY, t(8;21)	ITD
58	49	M	M3	46;XY, t(15;17)	ITD
64	48	M	M4	46;XY, inv(16)	Asp 835 Tyr
69	47	M	M4	46;XY, inv(16)	Asp 835 Tyr
80	34	M	M4	46;XY, inv(16)	Asp 835 Tyr
83	39	M	M2	46;XY, t(8;21)	Asp 835 Tyr
91	37	M	M2	46;XY, t(8;21)	Asp 835 Tyr
96	38	F	M6	46;XY	Asp 835 Tyr
108	35	F	M5	46;XY	Asp 835 Tyr
116	31	F	M1	46;XY	Asp 835 Tyr
118	33	F	M4	46;XY	Asp 835 Tyr
121	25	M	M2	46;XY, inv(16)	Asp 835 Tyr
128	45	F	M6	46;XY	Asp 835 Tyr

**Table 2.** Oligonucleotide primers used in PCR analysis.

		Primer sequences	Fragment size (bp)
	F	5′-TCTGTTTCATCGCTGAGTGAC-3′	
ITD			396
	R	5′-AGCCTTGAAACATGGCAAAC-3′	
	F	5′-CCAGGAACGTGCTTGTCA-3′	
Exon 20			352
	R	5′-TCAAAAATGCACCACAGTGAG-3′	

F: forward; R: reverse.
